# Responsiveness and Adaptability of Healthcare Facilities in Emergency Scenarios: COVID-19 Experience

**DOI:** 10.3390/ijerph19020675

**Published:** 2022-01-07

**Authors:** Marta Łukasik, Anna Porębska

**Affiliations:** Faculty of Architecture, Krakow University of Technology, 31-155 Krakow, Poland; marta.lukasik@pk.edu.pl

**Keywords:** public health, healthcare facilities, hospitals, post-2020 hospital design, strategic decision making, risk management, disaster response

## Abstract

The COVID-19 pandemic revealed many vulnerabilities of the contemporary built environment along with limited preparedness and low efficiency in mitigating unexpected and unprecedented challenges. This article discusses the efficiency and responsiveness of basic hospital spatial layouts in three different scenarios: normal operation; the segregation of a large number of patients and still providing them with access to emergency healthcare, typical for a pandemic; and a sudden, extremely high number of admissions typical for compound disasters and terrorist attacks. A set of parameters and a method for general adaptability assessment (GAAT) that can be used as a tool in decision-making processes as well as evaluation of both existing facilities and the new models for resilient hospitals resulting from the experience of the pandemic are proposed. The paper emphasizes why factors among which adaptability, convertibility, and scalability should be at the very core of hospital development and management strategies. It also discusses new models of adaptable healthcare facilities that enable day-to-day operations to continue alongside a pandemic, and other emergency scenarios.

## 1. Introduction

The COVID-19 pandemic placed unprecedented stress on healthcare systems and healthcare facilities worldwide, in particular nursing homes, hospice homes, and hospitals. It revealed how even a modest rise in admission volume can overwhelm a hospital beyond its functional reserve. The consequences of physical and mental exhaustion of the healthcare workforce [1,2,3] and of limited access to healthcare in general (from prevention and basic diagnostics, to high-level diagnostics, to cancer procedures, to operations, to intensive care) [1] along the pandemic itself have already resulted in a reduction of life expectancy [4,5].

Especially in the early phase of the pandemic, hospitals were forced to adopt cross-cutting strategies that included the creation of buffer areas between wards, divisions between contaminated and non-contaminated areas, decontamination areas for healthcare workers, as well as the transformation of acute care spaces (i.e., those already equipped with advanced systems). The lack of scientifically validated data or evidence-based protocols [1,6] resulted in ad hoc spatial re-organization while indications and solutions previously applied to tackle other diseases caused by viruses like Ebola and SARS, or bacterial epidemics such as tuberculosis, turned out to be inadequate for COVID-19 [1,6,7].

In order to overcome the saturation of spaces in existing hospitals, two main strategies have been applied [1,8]. One is providing temporary solutions with shipping containers, inflatable systems, tent structures, partition panels, etc.; the other features the transformation of non-sanitary buildings. After two years of the pandemic, some experts have assumed that strategies that convert large buildings such as stadiums or commercial and conference centers into temporary hospitals and other healthcare facilities will shift towards providing extra capacity within existing hospitals or in their very close proximity [8] while the design of new hospitals should be oriented towards enhancing their flexibility and, as a result, their resilience [1].

Flexibility in architectural design can be defined as a building’s ability to adapt to changed spatial requirements over the short, medium, or long-term perspective [9,10,11]. When applied to resilient healthcare facilities [10,11], it must correspond with the complexity and specific time-spans of transformation in medicine, technology as well as spatial organization of hospitals and healthcare facilities, as well as the variability of standards and procedures.

If we assume that the desired features of the functional-spatial layout of a modern hospital are adaptability, flexibility, and responsiveness, then these features can be guaranteed only by buildings with optimized configurations which will respond to sudden changes and shifting needs in real-time. An improperly designed hospital layout, just like an archaic building not adapted to new needs, may pose a direct threat to the proper course of treatment and even to a patient’s health and life since it limits the effectiveness of medical personnel [1,2,3,12,13,14]. Hospital design may impact patient safety, directly or indirectly, relating to both active or latent failure. Hence, analyzing the relationship between the functional and spatial arrangement of hospitals and their effectiveness in tackling the COVID-19 pandemic must become a part of the lesson to be learned from this dramatic global experience.

For centuries, hospitals had been places of refuge for the pilgrims, the homeless, and the sick. It was only at the turn of the eighteenth and nineteenth centuries that they became public institutions dedicated exclusively to medical treatment. Their architecture derived both from technical and artistic disciplines and was inevitably bound to change along with the development of medicine, building technology, technical equipment, and aesthetics [15,16]. The first in particular resulted in evolving views on treatment methods, which, as a consequence, changed the functional and spatial programs of hospitals [11,17,18].

Guideline systems based on the principles of how a hospital operates when medical procedures are performed, are intrinsically linked with medical technology [18,19,20,21]. Apart from changes in medicine (both treatment and technology), architecture and construction (new materials and regulations), there were the shifts in medical training and education (the general approach towards patients and their wellbeing included), and, more recently, changes in the environment and new challenges such as an aging society and diseases of affluence, in particular the increasing number of cancer cases.

Today, a common principle in hospital facility design is Evidence-Based Design (EBD) also referred to as Evidence-Informed Design or Research-Based Design augmented with a user perspective often referred to as Therapeutic Design or Supportive Design [1,22,23] as well as Design-for-all [24]. Such a broader approach to healthcare facility design was proposed by researchers focused on the influence of the environment on the course of treatment and convalescence [25,26] including from both personnel and patient perspectives [11,27,28,29,30,31]. These studies are based on the hypothesis that improvements in the design of space and the organization of the functioning of the environment can significantly contribute to, among other things, reducing the stress experienced by patients, improving the medical outcomes of therapeutic processes, increasing patient care effectiveness, reducing fatigue and stress felt by the staff, and minimizing the cost of medical care [1,3,27,30]. Studies indicate that aspects such as air quality, lighting, patient room design, and other interior design elements, in particular surface materials, can directly impact patient safety [28].

Along with innovative medical technologies and new, constantly redefined needs, new procedures that required more space have emerged. Until recently, the literature on healthcare facilities’ design, including their spatial layouts, has been dominated by studies that focused on highly specialized spaces and maximizing adaptation to strictly defined needs [15]. In Poland, a more interdisciplinary approach can be seen in publications by Niezabitowska [32] or studies by Tomanek [33] and Janowicz [34,35]. The latter is particularly interesting because Janowicz analyzed the possibility of using architectural measures to reduce hospital infections. Published before the outbreak of the COVID-19 pandemic, like most studies, it refers mainly to the most common bacterial infections that, until recently, seemed to pose the main threat in hospitals.

In terms of hospital spatial and functional organization, in recent years there has been a tendency to centralize services for entire hospital complexes, mainly in technical and economic terms as well as diagnostic and treatment services. This was parallel to the decentralization of secondary facilities or their outsourcing [36]. In older projects, there were tendencies to duplicate operating rooms and to design separate analysis or treatment rooms for pavilion wards [36].

In contemporary buildings, we can observe a tendency to enable all wards as well as open treatment departments placed in a compact block to use one shared diagnostic and treatment area on the lower floors (block/tower on a podium) [36,37]. The alternative is the horizontal configuration either along with one core (comb/finger-plan) or around internal courtyards (atrium/monoblock) [1]. Each type has its weak points. Some of them can be mitigated through site-specific modifications and/or advanced equipment. For instance, digitization and remote devices [1] can facilitate logistics in more complex layouts. However, as Capolongo et al. [1,10] and Brambilla et al. [11] emphasized, the pandemic has made the existing structural, organizational, and technological challenges of worn-out and obsolete healthcare facilities even more compelling. It increased the sense of urgency to redesign existing structures [10,11] and enhanced the need for new ones to be designed and built based on flexibility. Such an approach would allow us to accommodate current healthcare operations, adapt to time-sensitive physical transformations, and respond to contemporary and future public health emergencies [1,11].

Among recent papers that deal with the COVID-19 pandemic experience, there is the one by Lesan et al. [38] who described how improper space management can increase the risk of infectious disease transmissions. The authors highlight the role of environmental design on infection control focusing on the experience of health care workers.

Nioi et al. [3] emphasized that poor adaptability can lead to important organizational and structural flaws that have a negative impact on patients’ wellbeing and effectiveness of the healthcare provided. And yet, at the dawn of the third year of the global pandemic, reliable operational tools for assessing the level of flexibility of healthcare facilities that would support strategic decision-making and design processes are still work-in-progress.

Despite the plentiful amount of publications regarding responsiveness to the pandemic, few academic papers have addressed the flexibility of hospital facilities and the efficiency of their adaptation to the requirements of the COVID-19 outbreaks in terms of architectural design. Issues directly related to the problem can be found in Capolongo et al. [1,11], who developed a ‘decalogue’ of design strategies for resilient hospitals. The 2020 articles discuss hospital design issues such as strategic location factors: hospital networks, typology, the flexibility of functional and spatial programs, buildings’ user orientation, as well as modern installation and material solutions in the context of critical care. Capolongo also supervised the study by Brambilla et al. (2021) on an Optimized Flexibility Assessment Tool composed of nine evaluation parameters [1,11]: location; typology; flexibility; functional program, access, and flows; inclusiveness and EBD; Healthcare Territorial Network; patient safety; HVAC and Indoor Air Quality; innovative finishing materials and furniture; Healthcare Digital Innovation. Our proposal is similar in approach, but it is oriented towards different aspects and goals. Capolongo et al. indicated key strategies to be considered in the design of new resilient hospitals and in the re-organization of existing structures based on COVID-19 experience.

Our research is focused on defining the flexibility of healthcare facilities in the context of various disasters. The adaptability of different types of hospital layouts to the challenges resulting from the outbreaks of the pandemic caused by SARS-CoV-2 was compared with how those layouts operate under normal circumstances and how they can respond to other emergencies. As a result, the tool proposed in the paper is intended as a support for decision-making processes and it provides criteria suitable for emergency-related suitability assessment. It also shows how aspects critical for effective responsiveness are determined by the spatial layout of healthcare facilities.

## 2. Materials and Methods

The aim of this study was to identify which of the existing spatial layouts have the potential to become the basis for models of adaptable healthcare facilities that enable day-to-day operations to continue alongside a pandemic or other emergency scenarios, such as compound disasters or terrorist attacks. It is achieved by using a simplified assessment of the effectiveness of four basic functional-spatial layouts [36,37,39,40,41] as defined by circulation within the complex.

- cluster/campus
- comb/finger plan
- tower on a podium
- atrial.

The cluster layout, often referred to as the campus layout, also includes detached pavilion wards [36]. The comb layout, often referred to as the finger-plan, distinguishes itself by the so-called hospital street [39,40] that connects all wards placed on one or both sides. The atrial layout also includes monoblocks as defined by Prasad [39], i.e., singular structures with more than one internal courtyard. For each type, the minimum area that must be set aside for infectious patients and, consequently, the suitability of a given type of hospital in the disaster response system in the context of a COVID-19 pandemic is estimated.

The general effectiveness and adaptability of each spatial layout were assessed in three scenarios: normal operation; a scenario that requires the separation of a relatively large and constantly increasing (over a period of days, weeks, or months) group of patients while providing them with access to the diagnostic section, surgical blocks and operating rooms and intensive care units among others (pandemic, contamination, etc.); a scenario that requires the admission of a very large number of patients in a time as short as hours and/or days (natural and compound disaster, terrorist attack, warfare, etc.).

The following parameters were considered:

- patient flow
- personnel flow
- resource flow (materials, equipment, installations, etc.)
- airflow
- scalability
- convertibility
- modifiability
- comfort
- view and surroundings.

Flows, scalability, convertibility, and modifiability are parameters of adaptability [42]. Scalability refers to the ability of a structure to expand while maintaining the functioning of the system, convertibility to the ability of the structure or space to change while maintaining the functionality of the system, and modifiability to the ability to change the way a space is used while maintaining the functionality of the system. Versatility, the remaining parameter of adaptability [42], cannot be assessed at the level of the general spatial layout as it depends strongly on how a specific facility is equipped. Hence, it was not taken into consideration in this case.

Airflow efficiency was assessed taking into account the role of air circulation and ventilation systems in the spread of infectious diseases and other hospital infections [43,44]. Despite a negative pressure isolation room is a standard, the number of such rooms is usually limited. And even if it is not, as in the case of the well-equipped urban hospitals in the United States [6], their number was found to exceed the demand during the peaks of the subsequent waves of the pandemic. Moreover, even if more negative pressure rooms were provided, circulation routes still posed a risk even if not perceived as such [6].

Comfort and relation with the surroundings are crucial elements of the EBD approach. It has been noticed that they play an important role not only in everyday scenarios, but also during emergencies like the COVID-19 pandemic [1,3,29,30,31] as well as natural and compound disasters [45,46,47].

At the scale of spatial layout, comfort was estimated as the possibility of regulating the amount of natural light and fresh air entering the building, the legibility of the layout facilitating orientation, accessibility, etc.; in emergency situations, the possibility of visual contact with visitors who cannot enter the building but who, with appropriate precautions, may be admitted to the hospital premises, etc. were taken into consideration. The efficiency of the surroundings was estimated not only as treating it a space that can be designated as green areas and tribute to the patients’ wellbeing and faster recovery, but also as a reserve of land which can be used for temporary facilities in emergency situations.

A three-point rating scale applied is used in risk assessment [48]. The values assigned to each degree of effectiveness—effective (1), non-effective (0), anti-effective (−1)—emphasize the role the hospital building plays in the healthcare system. It can support the treatment process (1), it can have no significant positive impact on it, and/or its lack can be compensated by equipment elements or digital systems (0), and finally it can be represented as a risk in itself (−1). The same was assumed concerning adaptability: some layouts can change easily (1), while in others changes are either possible (0) or impossible (−1). Hence, bad solutions consume the efficiency of the building.

The assessment of the adaptability of different types of hospitals to changing emergency situations [Table 1, Table 2 and Table 3] has been based on an analysis of the existing procedures, the authors’ experience in designing hospital facilities, and many years of research on their functional-spatial layouts [36] as well as experience in designing resilient systems [45,49]. The results of the evaluation are summarized in Section 3.5.

## 3. Results

### 3.1. Public Health Facilities in Poland Prior to the COVID-19 Pandemic

The majority of existing polish hospitals—as in many other countries, developing and developed alike [1]—are technically outdated buildings. Their current state in Poland is rooted in the modern history of the country. The situation in which the Polish healthcare service found itself in the previous century required intensification of construction activities. Already before the Second World War, the number of hospital beds was insufficient. After the war, most of the buildings that survived were outdated and did not meet the requirements of modern medicine. Architects and doctors faced the difficult task of developing new models of healthcare facilities, from clinics, to hospitals, to sanatoriums, at the lowest cost possible.

The need to isolate the sick was apparent not only in the way the facilities were organized, but also in their location. Hospitals were built on fenced plots on the outskirts of cities and very rarely in city centers. Urban isolation was also visible within the complexes themselves, which were often free-standing pavilions with functionally independent cuboid forms. The Modernist doctrine had a significant impact on how the new facilities were designed and built assuming that the patient was treated by doctors and not by the hospital stay [50]. The hospital was a machine for treating the sick, a place for fighting disease. This was indicated, among other things, by the austere and ascetic interiors with a predominance of white, green, and blue colors, and visible medical equipment, testifying to the high level of technology and modernity. The complicated functional and spatial layout prevented easy orientation in space and created a sense of inferiority in patients. At the time, there was no focus on patient comfort, and they were placed in rooms with multiple beds and preventing a sense of privacy [50].

In Poland, the problem of the hospital building design was taken up in the 1970s. The most important publications of that period include Jachowicz’s paper [51] on the organization, technology, construction, and economics of hospitals, and Ostrowska’s pioneer interdisciplinary research [52], in which a method of hospital facility design based on cybernetics, IT theory and mathematical models were proposed. Outside the Iron Curtain, Rosenfield, in an article [53] published around the same time, dealt not only with a functional division of hospital wards and their specificity, but mostly with other factors that significantly affect the process of treatment and convalescence such as light and color or the hospital environment. The issue of natural light and illumination in hospitals was also discussed in an earlier publication by Hopkinson [54].

Since the 1970s, when many of the currently existing Polish hospitals were built and several scientific publications on this subject were written, there has been a long-term standstill in this field. It is also related to the fact that, according to 2018 Eurostat data [55], Poland was listed among the countries whose percentage expenditure on health care in relation to GDP (Gross Domestic Product) oscillated around 6.4%, while the average in the European Union is 9.9%.

Along with outdated and underfunded infrastructure come outdated and/or ineffective regulations. For instance, current Polish design solutions lack proper spaces for chemical and biological decontamination [56]. Decontamination devices are obligatory but their location is arbitrary [57].

Prior to the pandemic, the number of infectious wards in Polish hospitals dropped significantly [58]. Current regulations [56,57,59] for infectious disease wards do not sufficiently define the location of such a unit within the structure of the entire hospital. They also do not address the necessity of a separate entrance, the movement of patients and staff inside the hospital, or the transport of equipment and sterile materials in the circulation system to the extent corresponding with the challenges to be faced during the COVID-19 pandemic. An infectious disease ward, as well as an isolation room, can be located in the hospital space without providing a separate entrance from the outside that would ensure the segregation of patients, staff, equipment, and materials movement. An infectious patient may be operated on in an operating suite used by other non-infectious patients and it takes 24 h to decontaminate an operation room. There is also no requirement for an isolation room to ensure complete isolation of infected and non-infected patients, as well as staff, flows that revealed themselves critical [6]. There are no traces of taking this factor into consideration in the strategic decision-making processes taking place during subsequent waves of the COVID-19 pandemic (from early 2020 to late 2021) [59,60].

A common inconvenience that appears in Polish cases of Emergency Departments (ED) is the lack of a decontamination room, which is necessary in the case of an epidemic and is currently implemented ad hoc in the form of mobile structures [61]. Providing such areas is not even taken into consideration for Intensive Care Units (ICU) and Medical Imaging Units (MIU). Another oversight in current legislation and on the part of people who design healthcare facilities is the omission of airlocks in isolation rooms, which in effect turns them into single-bed VIP rooms with separate bathrooms.

### 3.2. Hospital Facilities and COVID-19 Response in Poland

In early 2020, during the first wave of the COVID-19 pandemic, the Polish Ministry of Health selected 12 infectious diseases hospitals (the so-called ‘single-named’ hospitals), to which patients requiring multi-specialist medical care with confirmed SARS-CoV-2 infection would be referred. During the second and third wave of the pandemic (that started in Poland in late September 2020 and early March 2021 respectively), the Ministry divided the strategy of fighting the coronavirus into three categories and limited the number of infectious diseases hospitals to 9.

The first category included hospitals in the so-called network of hospitals (more than 600). Based on the decision of the Voivode, these hospitals had to have isolation rooms. This decision entailed two possibilities. The first was the creation of an isolation ward at the expense of a regular ward—a function swap carrying with it a limitation in access to basic medical care for people not infected with the coronavirus. The second option was to create isolation beds in each ward. Each powiat hospital must have had a drive-through station that was independent and additional to the hospital structure. In most cases, these stations were implemented as portable, temporary tent structures.

The second category should secure treatment sites for patients with COVID-19 in infectious or observation-infectious disease wards in existing wards, on the basis of orders from provincial governors. This group consisted of 87 wards.

**Figure 1:**
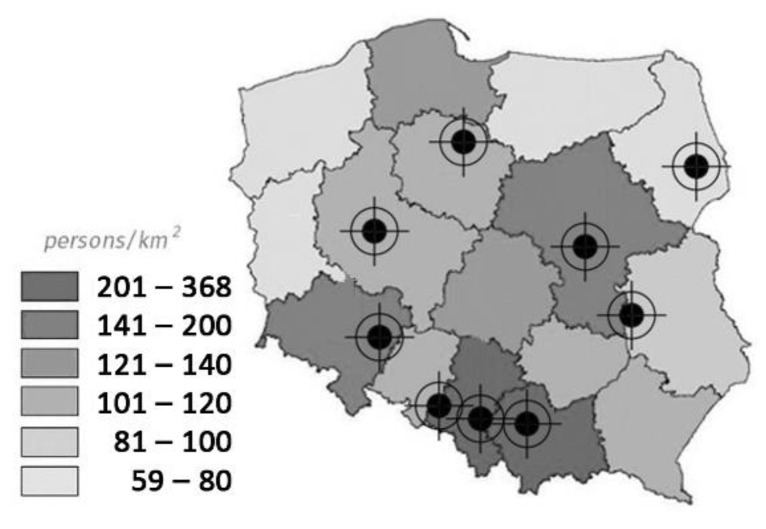
Map of Poland with marked Covidium hospitals, including population density in each voivodeship (region).

The third category consisted of 9 so-called Covidium hospitals that had, among others, the following units: internal medicine and infectious diseases, general surgery, obstetrics and neonatology, cardiology, intensive care, and orthopedics. Initially, it was assumed that not only COVID-19 infected patients, but also the non-infected, would receive care in such a hospital. Their locations set against the background of population density are presented in Figure 1.

During the fourth wave that started in late October 2021, the strategy no longer applied. Some temporary facilities remained operational, but not in all locations presented in Figure 1. Admissions of Coronavirus patients or detection of the local outbreak resulted again in canceling scheduled admissions and treatments which keeps worsening already limited access to healthcare.

A relatively low number of tests per 1000 people on one hand [62] and lack of post-mortem testing obscured the real scale of the pandemic in Poland until the annual report on mortality was published [5]. In a country with a population of 38 million, the number of deaths in 2020 exceeded the annual average for the last 50 years by more than 100,000 (477,000 to 364,000), while the death rate per 100,000 people reached its highest value since 1951. In 2020, 477,335 people died, a number that exceeded the figures for 2019 by 68,000. According to the official register of cases, less than 30,000 of those were related to COVID-19. What must not be underestimated is the general negative impact on accessibility to healthcare that partially results from inappropriate design and management of healthcare facilities unprepared for a disaster of such scale and length.

Unfortunately, when it came to assigning specific facilities to each of the three categories, the flexibility of their functional and structural layout was not considered, at least not according to official sources. As presented below, such an omission may have a significant impact on the efficiency of healthcare provided in the case of a regional- and/or national-scale emergency.

### 3.3. Adaptability of Hospital Facilities Depending on Their Layout

**Figure 2:**
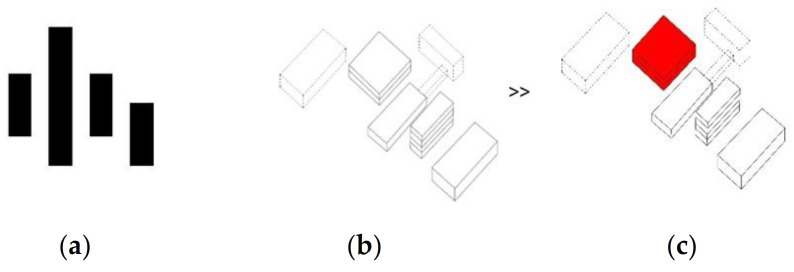
Free-standing detached pavilions: cluster/campus layout. (**a**) Planimetric scheme; (**b**) Axonometric scheme; (**c**) Axonometric scheme with the minimum area that must be dedicated to the infectious disease section indicated red.

**Figure 3:**
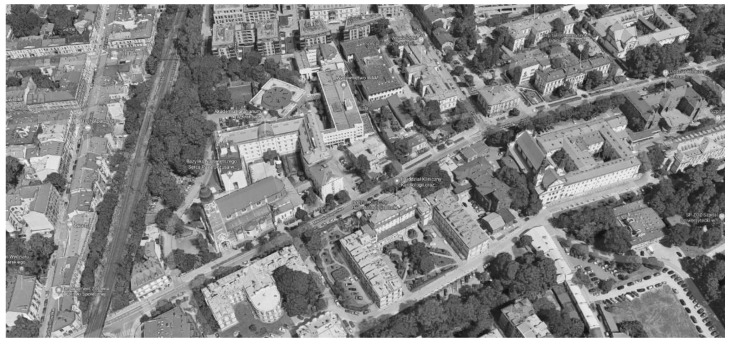
Cluster/campus layout: former clinical hospital in Kraków. Source: www.google.pl/maps (accessed on 1 September 2021).

**Figure 4:**
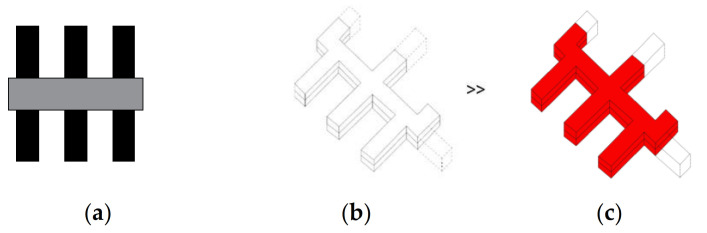
Comb layout (finger-plan). (**a**) Planimetric scheme; (**b**) Axonometric scheme; (**c**) Axonometric scheme with the minimum area that must be dedicated to the infectious disease section indicated red.

**Figure 5:**
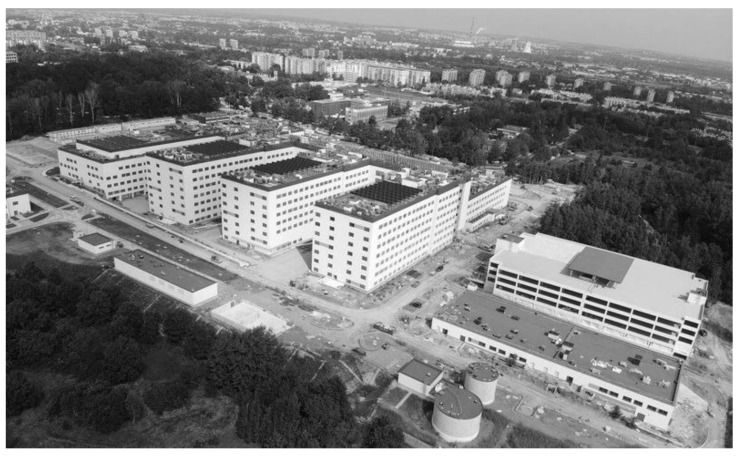
Comb layout (finger-plan) of the University Hospital in Prokocim, Kraków. Source: www.su.krakow.pl (accessed on 1 September 2021).

**Figure 6:**
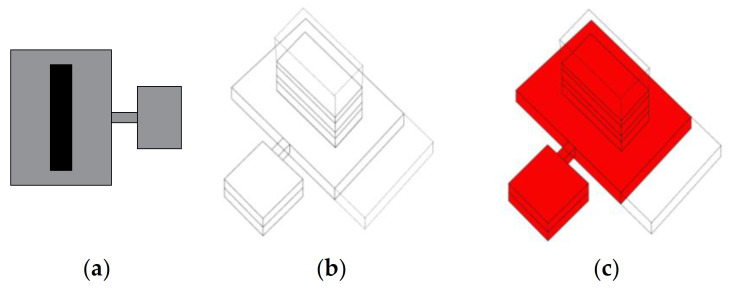
Tower-on-a-podium layout. (**a**) Planimetric scheme; (**b**) Axonometric scheme; (**c**) Axonometric scheme with the minimum area that must be dedicated to the infectious disease section indicated red.

**Figure 7:**
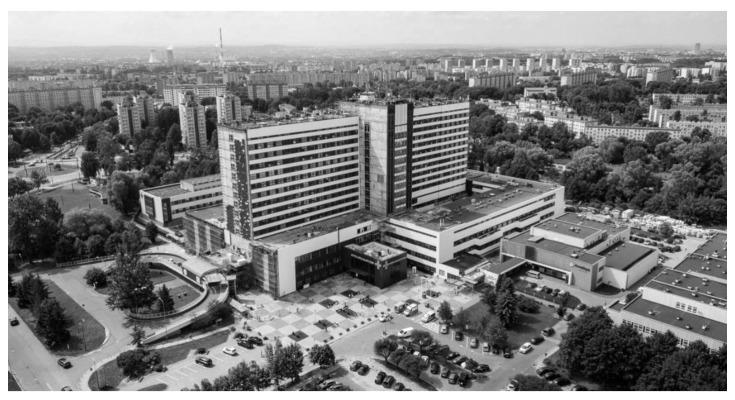
Tower-on-a-podium layout: Rydygiera Hospital in Krakow. Source: lovekrakow.pl (accessed on 1 September 2021).

**Figure 8:**
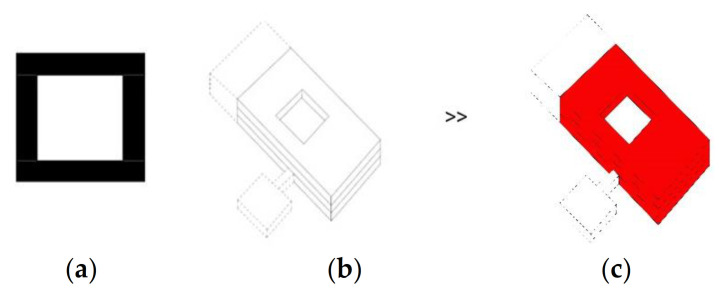
Atrial layout. (**a**) Planimetric scheme; (**b**) Axonometric scheme; (**c**) Axonometric scheme with the minimum area that must be dedicated to the infectious disease section indicated red.

**Figure 9:**
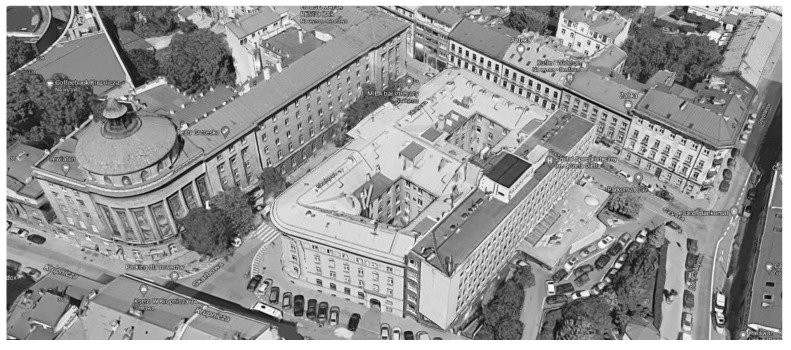
Atrial layout: Dietla Hospital in Kraków. Source: www.google.pl/maps (accessed on 1 September 2021).

The analysis of adaptability of different spatial-functional layouts of hospital facilities presented here focuses on four basic types: cluster/campus, comb (finger-plan), tower (block)-on-a-podium, atrial, and their general characteristics. The schemes of the four abovementioned typologies along with corresponding examples of hospitals in Kraków, Poland, the second-biggest city in the country and one of the nine locations marked in Figure 1, are presented in Figure 2, Figure 3, Figure 4, Figure 5, Figure 6, Figure 7, Figure 8 and Figure 9.

In the campus system (Figure 2 and Figure 3), the diagnostic and treatment units, the technology, and administrative units, and patient wards are often duplicated in each element, which significantly increases the cost of construction, use, and maintenance. Transporting a patient between buildings during the diagnostic process can be life-threatening, especially if the pavilions are detached. However, even if the wards are connected, the length of the passages and the complexity of the layout are an inconvenience in emergency scenarios. In comparison to other models (Figure 4, Figure 6 and Figure 8), this layout can be advantageous for long-term care and maternity or psychiatric hospitals because of natural advantages, like window views and a close visual connection with the surrounding environment.

The strong advantage of the campus layout is that it can easily be adapted to an epidemic situation, where one of the independent functional and spatial elements can be excluded from the standard operation and transformed into an infectious disease hospital with full access to diagnostic and treatment infrastructure.

In the comb layout, often referred to as the finger-plan, the main core provides a link between different wards (Figure 4 and Figure 5). This layout creates semi-open and partially private courtyards adjacent to the wards and potentially provides a visual connection with the natural environment. All paths that cross the core can increase infection rates by affecting both airborne and contact transmission routes. Even if one of the ward segments with its diagnostic and treatment areas is to be separated and turned into an infectious disease unit, it is extremely difficult to provide independent entrances to each of the zones, whether infectious or regular.

In the tower-on-a-podium layout presented in Figure 6 and Figure 7, functional and spatial separation is achieved by locating the wards on the upper levels and the diagnostic and treatment area in the large base [Figure 6]. This arrangement allows for relatively easy and fast circulation both for patients and staff inside the building, through the link connecting the two main parts of the hospital’s functional and utility section. The presented typology allows for the vertical extension of the ward section and the expansion of the footing, however, the structural expansion must be taken into account in the initial design phase. This arrangement provides optimal conditions for various installations, HVAC included. Due to the vertical arrangement of the wards, favorable viewing conditions and very good access to light are also ensured.

In this case, to the location of the wards in the vertical element, and the diagnostic and treatment areas at the base, functional and spatial clarity is achieved. The diagnostic base in this typology is very often extended to accommodate the necessary functions for which there was no room in the original building by adding additional blocks linked by footbridges with the main body. This arrangement allows for easy and fast circulation of both patients and staff inside the facility. It allows for the vertical extension of the wards and the expansion of the footing. However, it would be most prudent to consider the surplus structural expansions in the initial design phase. Due to the vertical layout of the ward unit, this type provides favorable views of the surrounding area and very good daylight access.

Whilst this layout is highly effective in terms of providing natural light and good ventilation as well as connecting the interiors with the surroundings, important to the well-being of the patients, it is extremely difficult to modify the way it operates and to separate any area in order to dedicate it safely to the infectious disease ward. It can be turned into a Covidium hospital to provide complete health care to patients with COVID or with other associated diseases.

The atrial layout consists of a single or multiplied interior courtyard surrounded by a continuous structure (Figure 8 and Figure 9). Circulation takes place around the atrium. This type is difficult to expand, assuming the original typology is to be preserved. The addition of functional elements is possible, for example, in the form of independent blocks connected to the main building by links.

Due to the peripheral movement of both patients and staff and the crossing of circulation routes at many points, it is difficult to propose a safe separation of the infectious disease ward. There is a high probability of crossing paths and spreading all types of nosocomial infections. For this reason, during a COVID-type outbreak, such hospitals should be excluded from admitting infected patients or the entire hospital building should be dedicated exclusively to COVID-19 treatment.

### 3.4. Adaptability of Basic Hospital Layouts to Different Scenarios

As mentioned above, the efficiency of different spatial layouts in responding was estimated for three different scenarios: everyday operation, a scenario that requires the separation of specific groups of patients (pandemic, contamination, etc.), and a scenario that requires a large number of patients to be admitted in a very short time (compound disaster, terrorist attack, warfare, etc.). As explained above, nine aspects were taken into consideration: the flow of patients, personnel, resources and air, modifiability, convertibility, scalability, comfort, and the surroundings. Since the assessment is general rather than detailed, a three-point, positive and negative scale was applied: effective (1), non-effective (0), and anti-effective (−1).

In the everyday operation scenario (Table 1), the tower-on-a-podium offers the most efficient flow of patients and personnel as well as convenient access to resources. This is due to the compact layout and circulation shafts usually located in the central part of the building. These are weakest in a cluster/campus layout due to the distance between elements and the need to duplicate many functions such as the entrance area, administration, supplementary rooms, etc. If the pavilion wards are not connected through indoor passages, this may be a life-threatening factor.

**Table 1:** Efficiency of different spatial layouts in everyday situation: effective (1), non-effective (0), anti-effective (−1).

	Clustered Layout	Comb Layout	Tower-on-a-Podium Layout	Atrial Layout
Flow of patients	−1	0	1	0
Flow of personnel	−1	0	1	0
Flow of resources	−1	0	1	1
Flow of air	1	1	0	0
Modifiability	0	0	1	1
Convertibility	1	1	1	−1
Scalability	1	1	−1	−1
Comfort	1	1	−1	0
View & surroundings	1	0	1	−1
	3	4	4	−1

In an everyday scenario, modifiability, convertibility, and scalability are important categories, due to the pace of development of medical technologies, requirements, and procedures. Comfort and environment are assessed here in terms of their impact on patient wellbeing (healing environment). In extreme situations (Table 2 and Table 3), their dual character was assessed, i.e., their usefulness in increasing the overall effectiveness of the system.

**Table 2:** Efficiency of different spatial layouts in an emergency scenario requiring separation of specific groups of patients (pandemic, contamination, etc.): effective (1), non-effective (0), anti-effective (−1).

	Clustered Layout	Comb Layout	Tower-on-a-Podium Layout	Atrial Layout
Flow of patients	1	0	−1	−1
Flow of personnel	1	0	−1	−1
Flow of resources	1	0	−1	−1
Flow of air	1	1	−1	−1
Modifiability	1	1	−1	−1
Convertibility	1	0	−1	−1
Scalability	1	0	−1	−1
Comfort	1	1	−1	0
View & surroundings	1	0	−1	−1
	9	3	−9	−8

**Table 3:** Efficiency of different spatial layouts in an emergency scenario requiring an extremely large number of patients to be admitted in a very short time (compound disaster, terrorist or military attack, etc.): effective (1), non-effective (0), anti-effective (−1).

	Clustered Layout	Comb Layout	Tower-on-a-Podium Layout	Atrial Layout
Flow of patients	−1	0	0	0
Flow of personnel	−1	0	1	0
Flow of resources	−1	0	1	1
Flow of air	1	1	0	0
Modifiability	0	0	1	0
Convertibility	0	1	1	1
Scalability	1	1	−1	−1
Comfort	1	0	−1	−1
View & surroundings	1	0	−1	−1
	1	3	1	−1

According to the results, the highest-rated typology in the situation of a threat of airborne diseases or viruses is the cluster/campus layout. It gives the possibility to separate individual blocks as infected zones without affecting the functioning of the others. Open spaces, which are a nuisance on a daily basis due to the discontinuity of the system, offer the possibility of adding temporary elements. The full functionality of such a system nevertheless requires that a pavilion can be dedicated to infectious patients, where it is possible to ensure the functioning of the surgical ward and the ICU, and access to diagnostic imaging.

The tower-on-a-podium layout, which is the most efficient in terms of flows under normal conditions, does not work well when there is a need for a separate area or ward for infectious patients. The communication and ventilation risers providing a transmission pathway favor the spread of viruses and other nosocomial infections [43,44].

In a scenario that requires the emergency admission of a large number of patients, the key problem is not only to ensure an adequate number of beds, but also the capacity for admissions, segregation (triage), and diagnosis. In this respect, the cluster/campus and comb layouts are more flexible. Again, the distribution and accessibility of specialist units is a decisive factor in assessing the efficiency of a particular hospital complex.

### 3.5. Towards an Adaptable Hospital Model

The results presented above are simplified guidelines in the assessment of the effectiveness of hospitals at the level of their spatial typology. In assessing the efficiency of specific buildings and hospital complexes, the following should be taken into account:

- safe and controlled patient flow with controllable capacity and accessibility
- easy flow of personnel with the provision of decontamination sluices (preferably in addition to staff areas or areas accessible to the public in the baseline scenario)
- layout of plant components, storage areas, and secondary facilities to allow for zoning and multi-point access: easy access to all resources in the normal operation scenario, 0 or +1 effectiveness in the emergency scenarios
- separate and hybrid ventilation systems
- functional variability that accounts for the efficiency of equipment and economic efficiency (balance between converting regular rooms into ICU rooms and multiplicating ICU rooms)
- possibility of remodeling while maintaining the functionality of the other elements of the system
- expandability, including ad-hoc expansion using temporary elements and container modules.

Should even such a general assessment had been made during the decision-making process, the location of the so-called single-named (Covidium) hospital in Kraków could have been different.

## 4. Discussion

### 4.1. Towards Resilient Hospital Models

All architectural creativity seemingly gives a permanent effect. The layout of a hospital is temporary and is determined by the circumstances of the moment, based on an active process of embedded intelligence and potential change. In a material sense, hospitals may remain in the urban space for years but their usability passes with the changing needs of their users. Therefore, a skillfully created hospital space should anticipate the challenges of the future. The transience associated with physical operation can be stopped, but the gradual obsolescence of utility associated with changing user needs and progress in medicine will be difficult to overcome.

As presented above, hospitals built as a cluster/campus system with the possibility of adaptation of one of the independent buildings for infectious disease ward are in a relatively comfortable situation in comparison to hospitals which are built as compact structures, in which there is a necessity of the separation the infectious disease ward within a functioning structure. The question is how to correctly locate these wards and how to separate them from other hospital wards so as to minimize nosocomial infection spread risk:

- The design of an Emergency Room (ER) type pavilion in close proximity to the main hospital building would result in such units being able to act as a completely self-sufficient ER and diagnostics unit for infectious cases in the event of an epidemic. At other times, they could be used as regular treatment wards. It should be remembered that in places where ‘infected’ and ‘healthy’ paths intersect, safety measures should be applied—including capsules and airlocks that protect against the spread of infection.
- Newly designed hospitals should allow the conversion of one or more wards into infectious disease wards in the case of an epidemic or other emergency. It is necessary to plan them in such a way that would allow a complete functional separation of the infectious disease ward from the rest of the hospital without crossing the paths of patients, staff, and supplying materials.
- The Hospital Emergency Ward, to which, as a rule, all patients are sent for unscheduled treatments, designed for larger units, could—in the case of a pandemic—play the role of a self-sufficient unit for admissions and diagnostics.
- A paved or a green area on the hospital property with adequate utilities can be dedicated to temporary extensions (modular systems, containers, tents, etc.). It can be connected to an existing unit.
- Supplementary areas in ICU, MIU, and surgical blocks should allow providing emergency by-pass routes in case of emergency.

Even before the pandemic, there was a growing recognition that buildings and public spaces, in particular those in the areas under threat of natural disasters, should be more flexible [63]. Today, a hospital is one of the most complex institutions, and shaping its architecture is a complicated and responsible task. That is why usability, transparency, and flexibility become a priority in creating health space. It follows the change that occurs in medicine, but at the same time, it becomes more and more patient-friendly. Such is the vision by Ab Rogers Design. ‘Living Systems’ won the Wolfson Economics Prize 2021, the second-largest economics prize after the Nobel Prize, for reimagining hospital design [64]. In the proposal, the wards are buried in vertical gardens and the hospital is surrounded by public spaces: an in-house marketplace of local produce, a rooftop urban farm, and pocket gardens between wards manned by ‘hosts’ whose job is to look after patients and their visitors. The project embodies the paradigm as the building as a ‘third career’, but it does not address the challenges among which the global pandemic or a limited budget.

The model proposed by HKS Architects [42] was based on the COVID-19 pandemic. The FleXX Hospital: Surge Scenario is a modified atrium/monoblock layout with multiplied circulation shafts. When the virus is detected in the population, one of these shafts becomes the dedicated entrance and circulation hub, separating flows of infected and virus-free patients. In the first phase of an outbreak, a ward is sub-divided to segregate the infected patients. As the case number increases, the area dedicated to the treatment of infected patients expands accordingly.

Unfortunately, this model focused mainly on the number of beds to be dedicated for infected patients and their separation within the structure of the hospital. Strategies for asymptomatic patients [65] and the length of the journey through the triage system [66] to the infectious ward to intensive care units and operation rooms are not revealed. Unless rooms to be converted into infectious wards are ICU rooms, and a surgical block and a diagnostic center, including Medical Imaging Unit can be dedicated to infected patients only, either the spectrum of treatment is limited or infected patients must be transported to other wards nevertheless. As a result, subsequent parts of the hospital are either contaminated or dedicated to infected patients only and scheduled admissions of non-infected patients must be canceled.

Capolongo et al. [1] proposed a hybrid configuration characterized by a main body connected to support pavilions with dedicated access for emergency and logistic vehicles. In case of an infectious emergency, the independence of the buildings or the availability of autonomous internal utilities, allows us to separate different functional areas from the rest of the hospital system, without interrupting standard operations. Providing outdoor spaces is suitable for hosting possible temporary structures such as tents, tensile structures, or other modular solutions, which guarantee the connection to the hospital and the relationship to the driveways for ambulances and logistic vehicles. It is a model suitable for pandemic-like emergencies (cf. campus layout, Table 2), less efficient in other emergency scenarios.

A combination of the three models described above with our approach and the General Adaptability Assessment Tool (GAAT) proposed here would result in a more realistic and more resilient framework.

### 4.2. Applicability of the General Adaptability Assessment Tool (GAAT)

Evaluation of functional and spatial layouts is critical to the hospital’s development strategy. Based on an economic decision, which is interpreted by the demand resulting from health needs maps, it is possible to determine which hospital should be rebuilt, expanded, and which should be closed. Such studies would allow for determining functional and spatial programs and to avoid design and development mistakes.

The COVID-19 pandemic revealed that efforts should be made not only to provide new models for future resilient healthcare facilities but also to modernize existing ones and to adapt them to potential new threats. Care should be taken to create isolation rooms with a direct entrance from the outside, allowing the separation of different flows, both in terms of selective and emergency admissions. The correct location of an infectious disease ward should be selected based on current experience and while accounting for possible future situations and ventilation systems adjusted to eliminate the possibility of cross-infection. Small fixtures that can be direct sources of infection and disease spread, such as bathroom faucets, should not be overlooked. Replacing them with touchless faucets can not only improve the situation of bacterial transmission but also reduce water consumption. Analogous items here are light switches and doorknobs, where the use of copper as a bacteria-reducing material is of constantly increasing interest.

The study presented here assumes pragmatic actions and takes into account the limited possibilities of Polish healthcare services to finance new development projects. The architectural correction of the layout of the functional-spatial and organizational structure, as well as the determination of the further direction of changes, may cause a significant improvement in the functioning of a hospital unit. The General Adaptability Assessment Tool (GAAT) aids in determining the weaknesses of a structure that should be addressed first.

Even if not detailed enough to be used while defining the aims and goals of a modernization of a specific facility or a final project strategy, the GAAT allows for a rapid assessment of general adaptability and efficiency of a healthcare facility based on basic data that can be found in any public register. Such a tool, once validated and refined, can support decision-making processes in disaster response.

## 5. Conclusions

The general adaptability assessment tool (GAAT) proposed in the paper is based on nine parameters. Seven are regarding flexibility (flows of air, patients, personnel and resources, modifiability, convertibility, and scalability) and two are oriented towards Evidence-Based Design (EBS) and Design-for-all (comfort and the surroundings). Each parameter is evaluated using a three-point ranking scale typical for risk assessment, in this case: effective, non-effective, anti-effective that correspond with 1, 0, −1 value. The tool is applied to analyze adaptability and responsiveness of four basic spatial layouts of hospitals: campus (cluster layout with detached pavilions), comb (often referred to as finger-plan), atrial, and tower-on-a-podium. The efficiency of each layout is estimated in three different scenarios.

The differences between efficiency in everyday use and two different emergency scenarios (pandemic and compound disaster) suggest that new models for resilient hospitals should learn from the COVID-19 pandemic but also look beyond this experience in order to respond properly to future challenges.

The tool can be applied for the evaluation of adaptability, flexibility, and efficiency of existing healthcare facilities as well as newly proposed models. Due to its basic and simplified character that requires easily accessible data, it is intended as a support for decision-making processes. It can be used as an introduction to site-specific research and as a multi-criteria analysis (MCA) matrix.
